# Cardioembolic Stroke Due to Prosthetic Valve Endocarditis Caused by *Candida parapsilosis*: A Case Report

**DOI:** 10.1155/2024/5581547

**Published:** 2024-11-09

**Authors:** Mohammad Nasser Affas, Yamane Chawa, Mohammad Salem Khalil, Sham Alkodmani

**Affiliations:** Department of Internal Medicine, Hamad Medical Corporation, Doha, Qatar

**Keywords:** *Candida parapsilosis*, cardioembolic stroke, prosthetic valve endocarditis

## Abstract

Embolic stroke due to prosthetic valve endocarditis (PVE) caused by *Candida parapsilosis* is a rare and serious complication. Successful management requires a combination of medical and surgical approaches. We present a case full of complexities in diagnosing and managing *Candida* PVE, emphasizing the importance of a multidisciplinary approach. A 50 year-old male presented to the emergency department with vertigo and low-grade fever and was found to have cerebellar stroke likely from the cardioembolic origin, and the patient had a history of uncontrolled diabetes and double prosthetic valves. The diagnosis was challenging and required transesophageal echocardiography (TEE) which showed two vegetations attached to the mitral valve prosthesis. The management involved antifungal therapy, but surgery was hindered by financial issues. The patient was considered for the AngioVac vegetation aspiration system due to persistent fungemia. Eventually, surgery was not performed, and the patient was discharged with a plan for long-term suppressive antifungal therapy.

## 1. Introduction

Fungal endocarditis (FE) accounts for less than 2% of infective endocarditis (IE) cases [1]. *Candida*, particularly *C. albicans* and *C. parapsilosis*, is identified as the most common cause of FE, with prosthetic valve endocarditis (PVE) due to *Candida* species being relatively uncommon, as an 11-year retrospective observational study from a quaternary cardiac referral institute in India found that around 25% of PVE cases were caused by *Candida* species [2].

*Candida parapsilosis* possesses various virulence factors, one of which is the capacity to create a biofilm on diverse medical devices and prosthetic materials [3].

Although the published data about IE in Qatar is limited, one study was published in 2019 showed that 19% of the cases were in association with prosthetic valves. The mitral valve was involved in 47%, C. parapsilosis was isolated in 2%, stroke occurred in 9% and a combined medical and surgical approach was possible in only 16% of the cases [4].

## 2. Case Presentation

A 50 year-old Nepalese male with a history of mitral and aortic valve replacements due to rheumatic heart disease presented to the hospital after experiencing vertigo. He had been on warfarin therapy for heart failure with reduced ejection fraction (LV EF 44%) and type 2 diabetes mellitus.

**Figure 1:**
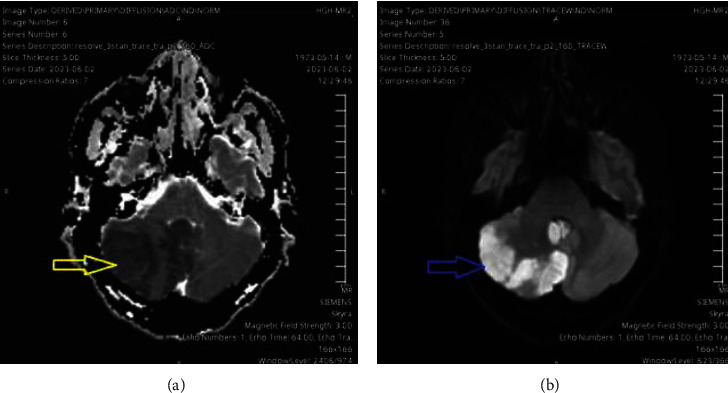
MRI of the head showing the right posterior inferior cerebellar area of diffusion restriction with involvement of the right tonsil: (a) apparent diffusion coefficient (ADC) and (b) diffusion-weighted imaging (DWI).

**Figure 2:**
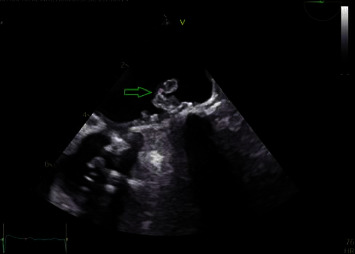
Transesophageal echocardiography, midesophageal four-chamber view, showing mitral valve vegetation.

Approximately 3 days prior to admission, the patient had discontinued warfarin, which led to an ischemic stroke in the posterior inferior cerebellar artery territory (Figure 1). Blood cultures obtained on admission due to a single febrile spike revealed *Candida parapsilosis* in two bottles. The ophthalmological evaluation showed multiple Roth spots in the left eye but no signs of endophthalmitis. Transesophageal echocardiography (TEE) confirmed the presence of vegetation on the mitral valve prosthesis sewing ring, measuring 1.5 × 0.8 cm and 0.3 × 0.4 cm (Figure 2).

Based on these findings, a diagnosis of *Candida parapsilosis* PVE was made. The patient was initiated on anidulafungin empirically and transferred to the Heart Hospital. After consultation with the infectious disease team, anidulafungin (IV 100 mg daily) was continued, and fluconazole (IV 800 mg daily) was added. Given the complexity of the case, the multidisciplinary team (MDT) recommended vegetation aspiration via the AngioVac technique.

**Figure 3:**
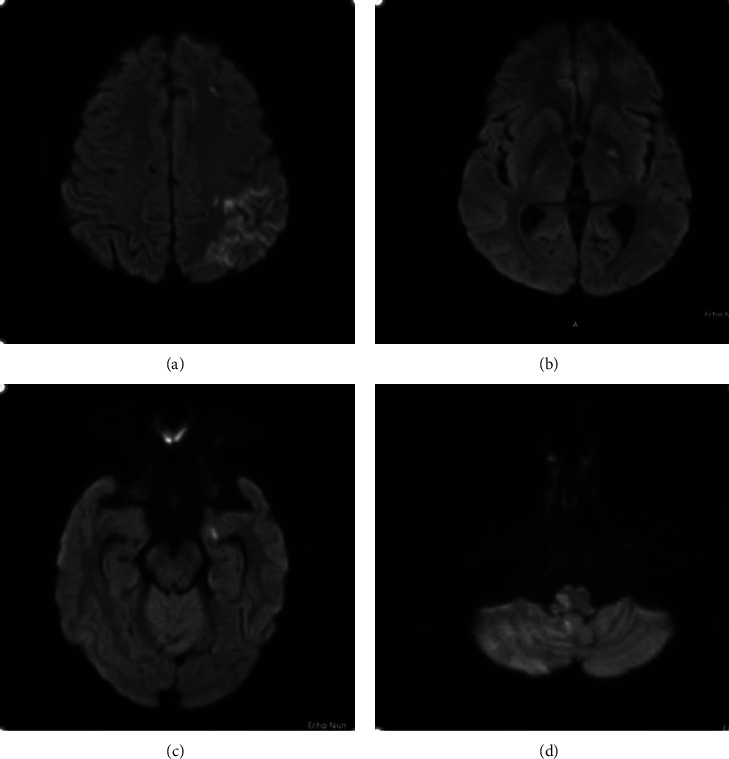
MRI head diffusion-weighted imaging showing diffusion restriction in the following areas: (a) right frontal and parietal lobe, (b) right internal capsule, (c) right temporal lobe, and (d) left medulla.

Two weeks after the presentation, the patient underwent the AngioVac procedure. During the procedure, wire manipulation caused the vegetation to be displaced, raising concerns about embolization. However, angiographic studies showed no evidence of dislodgement, and the patient remained neurologically stable. Unfortunately, a diffusion-weighted MRI on the same day revealed multiple recent infarctions (Figure 3).

Despite 6 weeks of IV antifungal therapy, blood cultures remained positive for *Candida*, prompting another MDT discussion. Double valve replacement surgery was recommended but could not be performed due to financial constraints. Follow-up echocardiography performed 6 weeks later showed small residual vegetations. The patient was eventually stabilized and transitioned to long-term oral fluconazole therapy (600 mg daily) for at least six months until surgical options could be revisited.

Six weeks after discharge, the patient was seen in the clinic, he was doing well and still on oral fluconazole therapy.

## 3. Discussion

FE constitutes approximately 1%–3% of all cases of IE, presenting significant challenges in clinical management due to its high morbidity and mortality rates exceeding 70% [5]. *Candida* species are the primary culprits, responsible for over half of FE instances [6]. A recent systematic review identified *C. albicans* and *C. parapsilosis* as the most prevalent species causing FE [7].

PVE attributed to *Candida* is relatively rare, comprising only 5% (4/74) to 10% (22/223) of all PVE cases [8]. *Candida parapsilosis* complex, typically colonizing the gastrointestinal tract, skin, and oropharynx, poses a risk primarily in individuals with prosthetic valves (57.4%), intravenous drug use (IVDU; 20%), intravenous parenteral nutrition (6.9%), abdominal surgery (6.9%), immunosuppression (6.4%), broad-spectrum antibiotic use (5.6%), and prior valvular disease (4.8%) [9]. Other risk factors include diabetes mellitus [10], of which our patient had uncontrolled diabetes (HbA1C 7.9%) and dual prosthetic valves.

*Candida* PVE typically manifests as a late complication (median time: 8.9 months) following valvular surgery, with nearly half of patients having a history of IE. In one case series, 58% of patients experienced late-onset PVE, occurring at 26 months [11]. In our case, the stroke, due to cerebral embolization, led to the diagnosis of PVE. While ischemic stroke complicates 8%–11% of IE cases [12], it shaped our management by prompting more extensive diagnostics. TEE revealed vegetations that TTE failed to detect, consistent with its higher sensitivity for PVE [13]. Our patient's clinical trajectory mirrors studies where cerebral embolization was a frequent manifestation of fungal PVE [11].

According to a systematic literature review from 1997 to 2014 and an analysis of 29 cases of *Candida* endocarditis, both native valve endocarditis (NVE) and PVE are biofilm-related diseases with common pathogenetic pathways. These often involve intestinal translocation of *Candida* species and transient candidemia, which may lead to fungal colonization and biofilm formation on the valve. The significance of biofilm formation is heightened in PVE, where the type of prosthesis affects disease onset, and antibiofilm treatments can significantly reduce mortality [14].

Regarding treatment, we initiated dual antifungal therapy based on susceptibility results and proposed double valve replacement surgery, in line with Infectious Disease Society of America (IDSA) guidelines advocating a combined antifungal and surgical approach for *Candida* PVE management [15]. Many patients, due to frailty, advanced age, or chronic illnesses, are deemed unsuitable for surgical valves [16]. Although surgery was initially recommended, financial constraints prevented it. Persistent fungemia led to the consideration of the AngioVac procedure for vegetation aspiration, a method gaining popularity for left-sided masses despite FDA approval only for right heart cases [17]. Unfortunately, the procedure was complicated by vegetation displacement, potentially increasing the risk of embolic events.

Literature supports that embolic complications are common in left-sided IE, with brain MRI detecting embolization in up to 80% of cases, half of which are subclinical [18]. In our case, the MRI revealed several new infarctions postprocedure, raising the question of whether alternative interventions such as valve replacement surgery would have been more appropriate.

## 4. Analysis of AngioVac Procedure

The AngioVac system has emerged as a viable alternative to surgery in treating high-risk patients with tricuspid valve/prosthesis and intravenous leads for permanent rhythm devices [19, 20]. Recent studies have demonstrated its safety and efficacy in diverse scenarios, primarily involving the venous system and right heart chambers [21, 22]. Although the goal of the procedure is not to fully eradicate the infection, the primary aim is to debulk vegetation, reducing the embolic risk and microbial burden, and thereby improving the effectiveness of antifungal therapy [20].

Treatment of aortic or mitral prosthesis endocarditis using the AngioVac system has been reported, with positive outcomes when used in combination with cerebral embolic protection devices [15, 23, 24]. In cases like this one, a floating mass on a left-sided valve presents a significant embolic risk, typically warranting surgery. However, frail patients, such as ours, may not tolerate conventional surgery, which involves cardiopulmonary bypass and cardioplegic arrest, making the minimally invasive AngioVac procedure a valid alternative when valve regurgitation is minimal.

Gerosa et al. described the use of the AngioVac system via a transapical approach for a mass on the ventricular side of a mitral bioprosthesis [17]. This approach was extended in treating IE involving both the mitral and aortic prostheses in other cases. The risk of vegetation embolization during the procedure remains a concern, particularly in left-sided masses. To mitigate this risk, some teams have used cerebral embolic protection devices during the procedure [23, 24], as was considered in this case. The need for precise imaging, especially real-time TEE, is crucial to guide the procedure and avoid complications, such as damage to the mitral valve subvalvular apparatus [17].

Though the patient experienced complications, including multiple cerebral embolizations postprocedure, the AngioVac system remains a promising option for high-risk patients. The decision to forego conventional surgery in favor of AngioVac reflects a growing trend toward minimally invasive solutions for managing complex infections in fragile patients.

## 5. Long-Term Management

Lifelong chronic suppressive therapy with daily oral fluconazole (6–12 mg/kg) is recommended for *C. parapsilosis* PVE patients who are ineligible for valve replacement, provided the *Candida* isolate is susceptible. The necessity for long-term suppressive therapy postvalve replacement remains debated, though many clinicians advocate for it to mitigate relapse risks. Relapse rates for *Candida* PVE are notably higher (29%) than for *Candida* NVE (5.3%) [25, 26].

In this case, the patient was discharged on suppressive oral fluconazole therapy.

## Data Availability

The data used to support the findings of this study are available from the corresponding author upon request. The data are not publicly available because they contain information that could compromise the privacy of our patients.
