# Corrigendum to “Cardioembolic Stroke Due to Prosthetic Valve Endocarditis Caused by *Candida parapsilosis*: A Case Report”

**DOI:** 10.1155/crdi/9872490

**Published:** 2025-08-18

M. Affas, Y. Chawa, M. Khalil, S. Alkodmani, “Cardioembolic Stroke Due to Prosthetic Valve Endocarditis Caused by *Candida parapsilosis*: A Case Report,” *Case Reports in Infectious Diseases* 2024 (2024): 5581547, https://doi.org/10.1155/2024/5581547.

In the article, the caption for Figure 1 is incorrect. The correct caption for Figure 1 is shown below:

We apologize for this error.

## Figures and Tables

**Figure 1:**
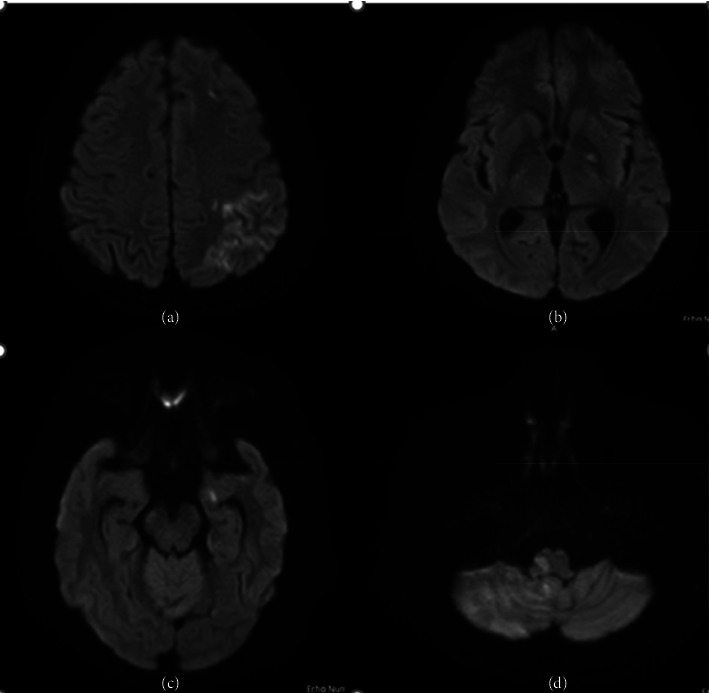
MRI head diffusion-weighted imaging showing diffusion restriction in the following areas: (a) left frontal and parietal lobe, (b) left internal capsule, (c) left temporal lobe, and (d) right medulla.

